# A low-cost, 3D-printed open-source platform for acute brain slice electrophysiology

**DOI:** 10.1016/j.ohx.2026.e00813

**Published:** 2026-07-04

**Authors:** Younsoo BYUN, Hyunjun Noh, Sung-Han Rhim, Jihyun Noh

**Affiliations:** a Department of Science Education, Dankook University, Yongin 16890, Republic of Korea; b Department of Mechanical Engineering, Dankook University, Yongin 16890, Republic of Korea

**Keywords:** Brain slice electrophysiology, Field recording, Hippocampus, Additive manufacturing, Open-source hardware, Low-cost instrumentation

## Abstract

Brain slice electrophysiology is a widely used approach for investigating synaptic physiology, network activity, and pharmacological responses. However, the high cost of commercial recording chambers and perfusion systems restricts accessibility in resource-limited laboratories. Here, we describe the design, fabrication, and electrophysiological validation of a fully integrated, open-source field recording platform for acute brain slice electrophysiology. The system comprises three independently assembled modules: a submerged recording chamber, a tissue-positioning stage, and a suction assembly, all fabricated via FDM (Fused Deposition Modeling) 3D printer using PLA (Polylactic Acid) filaments at a total material cost of approximately 1.23 USD. The platform was validated using acute hippocampal slices from adult male C57BL/6N mice. Field excitatory postsynaptic potential recordings from the CA3-CA1 Schaffer collateral pathway demonstrated high-fidelity signals and reproducible stimulus–response relationships, alongside stable 15-minute baselines. Thermal characterization confirmed uniform perfusate distribution and rapid temperature equilibration within the chamber bath. All design files and assembly instructions are openly available, with the hardware design files released under the CERN-OHL-S-2.0 license and the documentation under a CC BY 4.0 license, enabling straightforward replication by the broader neuroscience community.

## Highlights

• Fully integrated 3D-printed recording platform at a material cost below 1.30 USD.
• Three-module PLA design: recording chamber, positioning stage, and suction assembly.
• Uniform perfusate distribution confirmed by infrared thermography within 3 min.
• Validated for fEPSP recordings in hippocampal CA3-CA1 pathway in acute mouse slices.

## Graphical abstract

**Figure:**
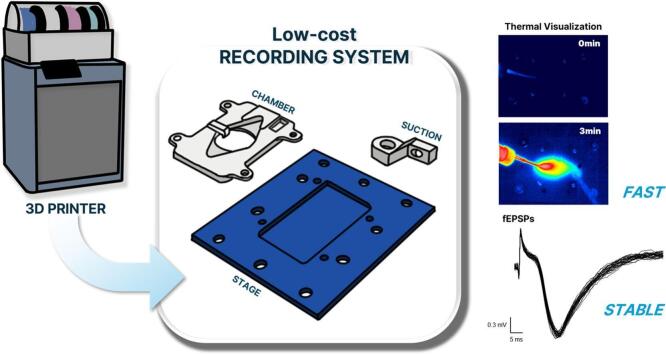

**Table:** 

**Specifications table**	
***Hardware name***	3D-Printed Open-Source Platform for Acute Brain Slice Electrophysiology
***Subject area***	Neuroscience
***Hardware type***	Biological sample handling and preparation
***Closest commercial analog***	Submerged slice recording chambers such as the RC-27 L (Warner Instruments, Hamden, CT, USA)
***Open source license***	CERN-OHL-S-2.0 (hardware); CC BY 4.0 (documentation)
***Cost of hardware***	1,750 KRW (1.23 USD)
***Source file repository***	*https://doi.org/10.5281/zenodo.21126914*

## 1 Hardware in context

Extracellular field potential recording from acute brain slices is among the most widely employed methods in cellular and systems neuroscience, enabling direct assessment of synaptic strength, long-term plasticity (LTP or LTD), pharmacological sensitivity, and network excitability under controlled ex vivo conditions [1], [2], [3], [4]. The preparation requires a recording chamber capable of maintaining slice viability through continuous perfusion of oxygenated artificial cerebrospinal fluid (aCSF) at near-physiological temperature while providing a mechanically stable substrate for electrode placement [1], [5], [6], [7]. Commercial systems fulfilling these requirements, such as submerged recording chambers supplied by Warner Instruments (Hamden, CT, USA), are reliable but carry acquisition costs frequently exceeding several thousand USD. These systems are often sold as components of larger instrument packages, including proprietary in-line heaters, temperature controllers, and perfusion manifolds, compounding the financial barrier for resource-limited laboratories and contributing to the concentration of this technique in well-funded institutions.

The rapid democratization of consumer-grade fused deposition modeling (FDM) 3D printing has substantially lowered the cost threshold for custom laboratory hardware fabrication [8], [9], [10], [11], [12], [13]. Prior open-source contributions have demonstrated the feasibility of addressing specific components of the brain slice recording setup. Hyde et al. demonstrated that polylactic acid (PLA), the thermoplastic most widely used in consumer FDM printers, is compatible with physiological buffers and does not adversely affect cell viability, providing an important biocompatibility precedent for PLA-based biological hardware [14]. Several groups have reported a 3D-printed recording chamber as an alternative to commercial designs [14], [15], [16]. While these contributions are valuable, each addresses an isolated element of the recording workflow; users seeking a complete field recording solution must source and adapt multiple independently designed components, introducing engineering variability, additional cost, and a barrier to reproducibility.

The present hardware addresses this gap by providing a fully integrated, single-platform solution comprising all components necessary for acute brain slice field recording: a sealed perfusion chamber, a tissue-positioning stage, and a suction assembly. All three modules are fabricated from standard PLA filament on a consumer-grade FDM printer and assembled exclusively with commercially available off-the-shelf fasteners, without requiring specialized tools, adhesives, or post-processing. The total material cost of approximately 1,750 KRW (1.23 USD) places this platform within reach of any laboratory with access to a desktop FDM printer, and the open-source design files enable adaptation to diverse experimental configurations.

## 2 Hardware description

The platform integrates three independently fabricated PLA modules into a cohesive field recording system for acute brain slice electrophysiology. The recording chamber incorporates a central bath sealed at its base by a 24 × 50 mm cover glass, secured with high-vacuum grease and four symmetrically positioned M3 Phillips pan head bolts that apply uniform compressive force to achieve a leak-free interface without adhesive. The bath cavity is shaped as a rounded rhombus to promote smooth, laminar distribution of incoming perfusate and eliminate stagnant zones at the periphery. An integrated right-angle bracket within the chamber body serves as a fixed anchor point for the ground electrode wire, maintaining continuous submersion within the bath solution and preventing flotation above the surface, thereby ensuring a stable electrical reference throughout the recording session. The suction assembly incorporates a commercially available needle-free adapter from a standard IV administration set as the primary suction conduit, engaged onto a 23 G hypodermic needle to maintain consistent suction height and thereby regulate bath fluid volume throughout recordings. The suction body features an open-slotted nut recess that permits tool-free vertical disengagement of the assembly, enabling rapid replacement of the hypodermic needle between preparations without disassembling the recording setup. All three modules were designed in Rhinoceros 3D, sliced with Bambu Studio, and fabricated on a Bambu Lab X1C printer using standard PLA filament, consuming approximately 22.45 g of material in total.

• The integrated platform enables high-fidelity extracellular field potential recordings from acute brain slices at a total material cost below 1.30 USD, substantially lowering the instrumentation barrier for resource-limited and early-stage neuroscience laboratories.
• The hardware design files are released under the CERN Open Hardware Licence Version 2 Strongly Reciprocal (CERN-OHL-S-2.0), and the accompanying documentation is released under a Creative Commons Attribution 4.0 International (CC BY 4.0) license, permitting reproduction, modification, and redistribution.
• The modular three-component architecture allows individual replacement of the chamber, stage, or suction assembly in the event of mechanical failure or biological contamination, without requiring full system reconstruction.
• The system is compatible with standard gravity-driven perfusion setups, conventional temperature controllers and in-line heaters, and off-the-shelf electrode manipulators, facilitating straightforward integration into existing electrophysiology workstations.
• Assembly requires no specialized tools, no post-print processing beyond press-fitting of standard hardware nuts, and no adhesives for the recording chamber seal, reducing technical overhead and enabling rapid maintenance during high-throughput recording sessions.

## 3 Design files summary

Design files for all three printed components are deposited in an open-access repository on Zenodo. The hardware design files (STP) are released under the CERN-OHL-S-2.0 license, and the accompanying documentation, including the print settings README, is released under a CC BY 4.0 license.

Repository: [Zenodo *https://doi.org/10.5281/zenodo.21126914*].

**Table:** 

**Design file name**	**File type**	**Open source license**	**Location of the file**
Stage_body.stp	STP	CERN-OHL-S-2.0	*https://doi.org/10.5281/zenodo.21126914*
Chamber_body.stp	STP	CERN-OHL-S-2.0	*https://doi.org/10.5281/zenodo.21126914*
Suction_body.stp	STP	CERN-OHL-S-2.0	*https://doi.org/10.5281/zenodo.21126914*
print_settings_README.txt	Plain text	CC BY 4.0	*https://doi.org/10.5281/zenodo.21126914*

**File descriptions:**

• ***Stage_body.stp:*** The positioning stage body is a single-piece PLA component designed to receive four M3 nickel-plated hexagon nuts in pre-configured recessed nut seats via press-fit or adhesive bonding. The upper surface provides a flat interface for seating the chamber body and the lower surface incorporates fixation geometry for attachment to a standard Faraday cage platform.
• ***Chamber_body.stp:*** The recording chamber body is a single-piece PLA component incorporating a rounded-rhombus bath cavity, bilateral inlet and outlet ports for perfusate flow, four threaded bolt channels at the corners for M3 Phillips pan head bolt-driven cover glass retention, an integrated right-angle bracket for ground electrode fixation, and a structurally reinforced, spatially isolated suction recess.
• ***Suction_body.stp:*** The suction assembly body is a single-piece PLA component featuring an open-slotted nut recess that permits tool-free vertical disengagement of the assembly and a lateral port for connection to the needle-free IV administration set adapter.
• ***print_settings_README.txt:*** A plain-text file documenting the Bambu Studio slicer settings used for all three components, including layer height (0.2 mm standard and initial), extrusion temperature (220℃), build plate temperature (60℃), nozzle diameter (0.4 mm), and filament specification.

## 4 Bill of materials summary

**Table:** 

**Designator**	**Component**	**Number**	**Unit cost**	**Total cost**	**Source of materials**	**Material type**
**S1**	Stage body (3D-printed PLA, 13.32 g)	1	239.76 KRW / 0.168 USD	239.76 KRW / 0.168 USD	Printed in-house (see Design files)	Polymer
**S2**	Hexagon nut (nickel-plated, M3)	4	40.00 KRW / 0.028 USD	160.00 KRW / 0.112 USD	Local hardware supplier	Metal
**C1**	Chamber body (3D-printed PLA, 4.18 g)	1	75.24 KRW / 0.053 USD	75.24 KRW / 0.053 USD	Printed in-house (see Design files)	Polymer
**C2**	High-vacuum grease (MOLYKOTE; DuPont; Wilmington, DE; USA)	0.3 g	162.80 KRW/g / 0.114 USD/g	48.84 KRW / 0.034 USD	Local laboratory supplier	Polymer
**C3**	Microscope cover glass (24×50 mm; Paul Marienfeld GmbH and Co. KG; Lauda-Königshofen; Germany)	1	88.94 KRW / 0.062 USD	88.94 KRW / 0.062 USD	Local laboratory supplier	Non-specific
**C4**	Phillips pan head bolt (stainless steel, M3 × 25 mm)	4	40.00 KRW / 0.028 USD	160.00 KRW / 0.112 USD	Local hardware supplier	Metal
**U1**	Suction body (3D-printed PLA, 4.95 g)	1	89.10 KRW / 0.063 USD	89.10 KRW / 0.063 USD	Printed in-house (see Design files)	Polymer
**U2**	IV administration set (needle-free adapter, catheter type)	1	436.00 KRW / 0.306 USD	436.00 KRW / 0.306 USD	Local medical supplier	Polymer
**U3**	Hypodermic needle (23 G, 25 mm)	1	32.00 KRW / 0.022 USD	32.00 KRW / 0.022 USD	Local medical supplier	Metal
**U4**	Socket head cap screw (stainless steel, M6×25 mm)	1	220.00 KRW / 0.154 USD	220.00 KRW / 0.154 USD	Local hardware supplier	Metal
**U5**	Hexagon nut (nickel-plated, M6)	2	100.00 KRW / 0.070 USD	200.00 KRW / 0.140 USD	Local hardware supplier	Metal
**TOTAL**				1,749.88 KRW / 1.226 USD		

Exchange rate applied: 1 KRW = 0.000702 USD.

## 5 Build instructions

All design files referenced below are available at the Zenodo repository listed in the Design files section. Component part numbers correspond to the Bill of materials summary.

**Safety note**: High-vacuum grease contains silicone oil. Avoid contact with eyes. The cover glass is fragile; apply only the minimum compressive force necessary when fastening the M3 bolts. No sharp tools are required for assembly.  

**Fig. 1:**
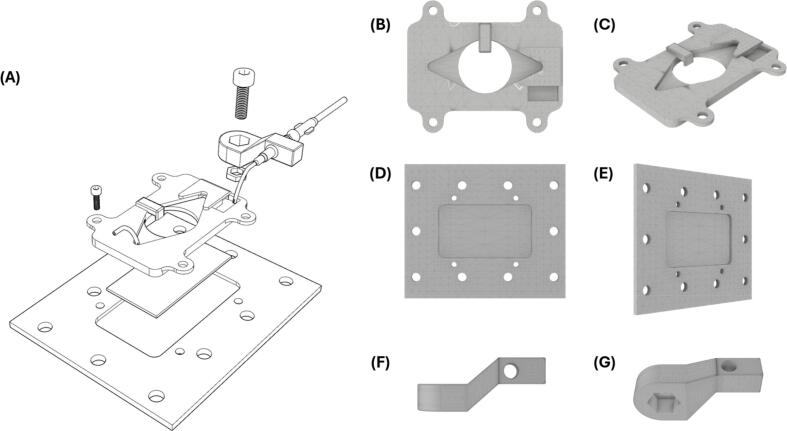
**Schematic design and component overview of the 3D-printed brain slice recording chamber system.** (A) Exploded assembly view showing the spatial relationship between the positioning stage, recording chamber, and suction module. (B ∼ G) Individual 3D-printed parts shown in top and perspective views, illustrating the stage body (B, C), chamber body (D, E), and suction-related components (F, G).

***3D printing of components (*** Fig. 1 ***):***

1. Download Stage_body.stp, Chamber_body.stp, and Suction_body.stp from the Zenodo repository.
2. Import each STP file into Bambu Studio (Bambu Lab; Shenzhen; China) or an equivalent FDM slicer.
3. Configure print parameters as specified in print_settings_README.txt: nozzle diameter 0.4 mm, standard and initial layer height 0.2 mm, extrusion temperature 220℃, build plate temperature 60℃. Keep all remaining parameters within the filament manufacturer's recommended range for PLA. These settings were selected to balance dimensional accuracy and layer adhesion for PLA; alternative slicer software may be used provided equivalent parameters are applied.
4. Slice and print each component individually on a consumer-grade FDM printer (Bambu Lab X1C; Bambu Lab; Shenzhen; China) loaded with 1.75 mm PLA filament (Bambu Lab; Shenzhen; China). No support structures are required, as all components were designed with self-supporting geometries. Expected filament consumption: stage body (S1) 13.32 g, chamber body (C1) 4.18 g, suction body (U1) 4.95 g.
5. After printing, confirm that all nut seats and bolt channels are free from stringing or layer artifacts. If necessary, clear residual filament from nut recesses using a 3 mm drill bit or a matching bolt rotated by hand.

**Fig. 2:**
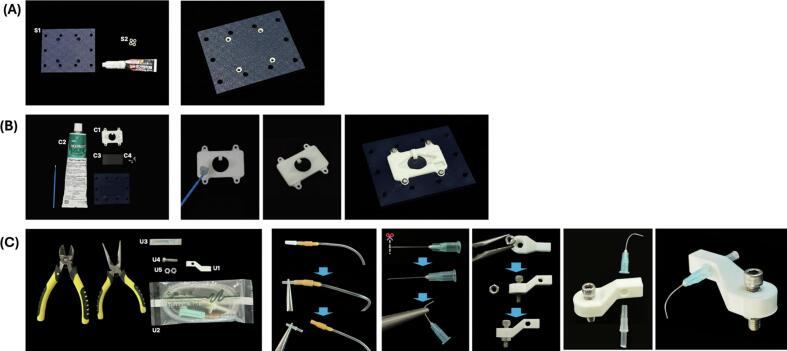
**Step-by-step assembly of the 3D-printed modules.** (A) Stage assembly using the printed body (S1) and M3 hexagon nuts (S2). (B) Chamber assembly with cover glass (C3) sealed by high-vacuum grease (C2) and secured by M3 Phillips bolts (C4), mounted on the chamber body (C1). (C) Suction assembly with an M6 bolt (U4) inserted through the suction body (U1). The first nut (U5) clamps the suction body onto the bolt, and the second nut (U5), positioned at the bottom, sets the height. A 23 G hypodermic needle (U3) and IV administration set adapter (U2) are attached to complete the assembly.

***Stage assembly (*** Fig. 2 ***A):***

6. Orient the stage body (S1) with the nut seats facing upward.
7. Press-fit one M3 nickel-plated hexagon nut (S2) into each of the four recessed nut seats until the nut is flush with the surrounding surface. The recessed nut seat geometry is designed to receive standard M3 hexagon nuts with a friction fit, eliminating the need for threaded inserts or heat-set hardware. For enhanced long-term mechanical stability, apply a small drop of cyanoacrylate adhesive to each nut seat before insertion and allow to cure before proceeding.
8. Verify that each nut is fully seated and correctly oriented by threading an M3 bolt into each nut seat by hand; the bolt should engage without resistance across at least three full turns.

***Chamber assembly (*** Fig. 2 ***B):***

9. Apply a continuous thin bead of high-vacuum grease (C2; MOLYKOTE High-Vacuum Grease; DuPont; Wilmington, DE; USA) to the entire perimeter of the recessed glass seat on the underside of the chamber body (C1). High-vacuum grease was selected over permanent adhesives to enable non-destructive disassembly for cleaning and cover glass replacement.
10. Place a 24 × 50 mm cover glass (C3; Paul Marienfeld GmbH and Co. KG; Lauda-Königshofen; Germany) onto the greased seat, pressing gently to ensure uniform contact across the full sealing surface.
11. Insert four M3 × 25 mm stainless steel Phillips pan head bolts (C4) through the corner channels of the chamber body (C1).
12. Thread each bolt (C4) into the corresponding nut (S2) in the stage body and tighten in a diagonal sequence (i.e., tighten opposite corners alternately) to apply uniform compressive force across the cover glass. The diagonal tightening sequence ensures even distribution of compressive force across all four corners, preventing localized stress concentration that could crack the cover glass. Finger-tight plus one quarter-turn is sufficient; overtightening risks cracking the cover glass.
  **Note:** Standard Phillips pan head bolts (C4) were selected to minimize procurement requirements, as they are available in most laboratory settings. Users who prefer tool-free assembly may substitute these with M3 winged (thumb) screws of equivalent length as an optional modification.
13. Inspect the cover glass-grease interface from below for continuity. Fill the chamber bath with distilled water and allow to stand for two minutes; confirm the absence of leakage before proceeding to use.

***Suction assembly (*** Fig. 2 ***C):***

14. Thread the M6 × 25 mm stainless steel socket head cap screw (U4) through the central bore of the suction body (U1) from the top.
15. Thread one M6 nickel-plated hexagon nut (U5) onto the bolt from below until it contacts the lower face of the suction body, and tighten by hand to clamp the suction body in place.
16. Thread a second M6 hexagon nut (U5) further down the bolt to the desired height position, then tighten both nuts against each other to lock the assembly at that height. A sufficient length of the bolt end must remain protruding below the second nut to engage the open-slotted nut recess of the chamber body (C1).
17. Insert the needle-free adapter of the IV administration set (U2) into the lateral port of the suction body (U1) until fully seated.
18. Attach a 23 G, 25 mm hypodermic needle (U3) to the needle-free adapter (U2).
19. To install the suction assembly onto the recording setup, lower the suction body (U1) vertically into the open-slotted nut recess of the chamber body (C1) until the protruding bolt end is positioned at the desired bath surface level. The open-slotted recess design enables tool-free vertical installation and removal, facilitating rapid needle replacement between preparations without disassembling the recording setup. To remove, lift the assembly vertically upward without rotation.

## 6 Operation instructions

**Safety note:** Isoflurane is a volatile inhalation anesthetic; use only in a well-ventilated area or with an appropriate anesthetic scavenging system in accordance with institutional safety guidelines. The 23 G hypodermic needle is a sharp instrument; handle with care and dispose of immediately in a designated sharps container after use. Carbogen (95% O_2_ / 5% CO_2_) is a compressed gas; handle and store in accordance with institutional compressed gas safety protocols.  

***Preparation of solutions:***

1. Prepare NMDG-based cutting aCSF and standard aCSF with the following compositions. NMDG cutting aCSF (in mM): NMDG 92, NaHCO_3_ 30, NaH_2_PO_4_ 1.25, KCl 2.5, D-(+)-glucose 25, MgSO_4_ 10, thiourea 2, Na-ascorbate 5, Na-pyruvate 3, HEPES 20, CaCl_2_ 0.5. Standard aCSF (in mM): NaCl 126, NaHCO_3_ 26, NaH_2_PO_4_ 1.25, KCl 3, D-(+)-glucose 10, MgSO_4_ 1.3, CaCl_2_ 2.4. Bubble both solutions continuously with carbogen (95% O_2_ / 5% CO_2_) for a minimum of 30 min before use. Verify pH (7.3 to 7.5) and osmolality (300 to 310 mOsm/kg) prior to slice preparation.
2. Chill the cutting aCSF to approximately 1℃ by submersion of the container in an ice bath.

***System setup:***

3. Mount the fully assembled recording chamber and stage onto the recording platform within a Faraday cage. Connect the gravity-driven perfusion line to the chamber inlet port and direct the tubing from the IV administration set adapter of the suction assembly to a waste reservoir as illustrated in Fig. 3A and B.

**Fig. 3:**
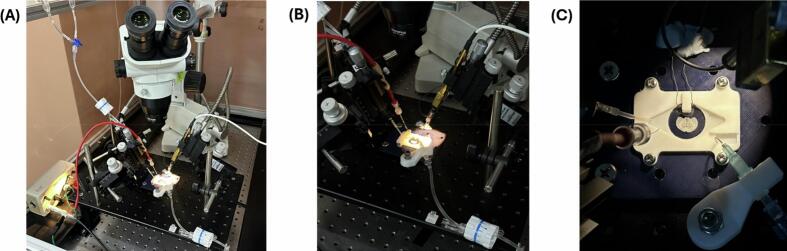
**Fully integrated experimental setup within a Faraday cage.** (A) Overview of the complete recording environment within the Faraday cage, showing the gravity-driven perfusion system, stage, and all associated electrophysiology hardware in their operational configuration. (B) Close-up view of the 3D-printed perfusion and recording system seated on the stage, with the gravity-driven perfusion line entering the chamber from above. (C) Top-down view of the recording chamber at higher magnification, showing the chamber bath with an acute hippocampal slice positioned on the coverslip floor during an active recording session.
4. Connect the in-line heater (SH-27B; Warner Instruments; Hamden, CT; USA) to the perfusion line and the temperature controller (TC-324B; Warner Instruments; Hamden, CT; USA) to the in-line heater and thermistor assembly (TA-29; Warner Instruments; Hamden, CT; USA).
5. Position the thermistor assembly at the chamber outlet site, proximal to the suction port, as illustrated in Fig. 4B.
6. Set the temperature controller target to 31.5℃ for electrophysiological recording. Activate the heater and perfusion flow with carbogen-bubbled aCSF and allow the system to reach thermal equilibrium for a minimum of 10 min before transferring brain slices. A transient temperature overshoot may occur during the initial warm-up period; this is attributable to the feedback response kinetics of the temperature controller and resolves within the equilibration period. Verify that the thermistor readout is stable at the target temperature before slice transfer.
7. Adjust the vertical position of the suction assembly within the chamber body to set the desired bath fluid level.

***Brain slice preparation:***

8. Deeply anesthetize adult male C57BL/6N mice (8 weeks old) with isoflurane (HANA PHARM Co., Ltd.; Seoul; Republic of Korea) and decapitate. Rapidly extract the brain.
9. Immediately immerse the extracted brain in ice-cold, carbogen-bubbled NMDG cutting aCSF and allow to chill for 1 to 2 min before slicing.
10. Prepare coronal hippocampal slices (350 μm) using a vibratome (COMPRESSTOME VF-210-0Z; Precisionary Instruments; Greenville, NC; USA).
11. Transfer slices to a holding chamber containing standard aCSF and incubate for a minimum of 2 h at 30℃ in a temperature-controlled water bath (Precision General Purpose Baths TSGP2S; Thermo Scientific; Waltham, MA; USA) before use.

***Brain slice transfer and electrode placement:***

12. Using a wide-bore transfer pipette, gently transfer a single acute hippocampal brain slice from the holding chamber to the recording chamber bath, as shown in Fig. 3C.
13. Allow the slice to settle and stabilize for a minimum of 10 min under continuous perfusion at 2 mL/min before initiating electrode placement.
14. Under visual guidance, position the stimulating electrode fabricated from chloride-coated silver wire in the Schaffer collateral region of hippocampal area CA3.
15. Position the recording electrode (borosilicate glass pipette, approximately 3 MΩ, filled with standard aCSF, mounted in microelectrode holder MEH3SW; World Precision Instruments; Sarasota, FL; USA) in the stratum radiatum of hippocampal area CA1.
16. Connect all electrodes to the amplifier (DAM 80; World Precision Instruments; Sarasota, FL; USA), stimulus isolator (ISO-Flex; A.M.P.I.; Jerusalem; Israel), and data acquisition system (Digidata 1440A; Molecular Devices; San Jose, CA; USA).

***Recording:***

17. Deliver test stimulation pulses via the pulse generator (Master-8; A.M.P.I.; Jerusalem; Israel) and confirm the presence of a clean fEPSP waveform in the stratum radiatum before proceeding. Adjust electrode position as necessary to maximize signal amplitude and minimize artifact.
18. Acquire a baseline recording of at least 15 min with test pulses at 60 μA delivered at 15-second intervals, averaging two consecutive responses per time point, to confirm signal stability prior to any experimental manipulation.
19. After recording, remove the slice using a transfer pipette, rinse the chamber bath with distilled water, and replace the hypodermic needle in the suction assembly with a sterile unit for the subsequent preparation. If biological contamination of the chamber body is suspected, disassemble by unfastening the four M3 bolts, remove the cover glass, and clean all surfaces with 70% ethanol before reassembly.

## 7 Validation and characterization

The performance of the 3D-printed field recording platform was validated across two independent experimental domains: thermal characterization of perfusate distribution within the recording chamber, and electrophysiological assessment of fEPSP signal quality in acute hippocampal brain slices. Together, these experiments were designed to confirm that the PLA recording environment supports both the physiological maintenance requirements and the signal fidelity requirements of submerged acute slice electrophysiology.  

**Fig. 4:**
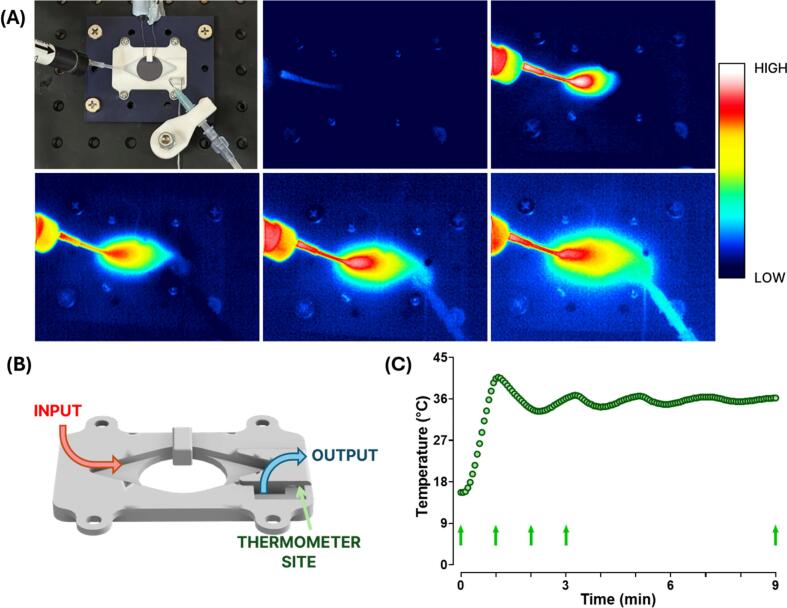
**Thermal characterization of perfusate circulation within the 3D-printed recording chamber.** (A) The leftmost panel shows a photograph of the actual recording chamber system during active perfusion; the remaining five panels show sequential infrared thermal images acquired at 0, 1, 2, 3, and 9 min after heater activation, illustrating the spatial propagation of heated perfusate through the chamber bath. The color scale represents relative thermal intensity (HIGH/LOW) rather than absolute temperature, as infrared imaging reflects surface radiance. (B) Schematic diagram of the recording chamber indicating the positions of the perfusion input, output, and thermistor assembly site at the pre-suction corridor. (C) Time course of bath temperature recorded at the thermistor assembly site, demonstrating rapid equilibration to the 36℃ target setpoint within approximately 3 min of heater activation and stable maintenance thereafter.

***Thermal characterization (*** Fig. 4 ***):***

To evaluate perfusate circulation dynamics, heated aCSF (target 36℃) was introduced into the recording chamber, which had been pre-filled with room-temperature solution (ambient temperature 16 to 16.1℃). This thermal contrast was used to visualize the spatial propagation of the heated solution via infrared thermal imaging (Testo 868; Testo SE and Co. KGaA; Titisee-Neustadt; Germany), with images acquired at 4-second intervals. Representative frames at 0, 1, 2, 3, and 9 min after heater activation were selected to illustrate the spatial and temporal progression of heated perfusate across the bath (Fig. 4A). Concurrently, bath temperature was monitored at the outlet site by a thermistor assembly (TA-29; Warner Instruments; Hamden, CT; USA) connected to the temperature controller (TC-324B; Warner Instruments; Hamden, CT; USA), positioned in the pre-suction corridor proximal to the suction port (Fig. 4B).

Infrared imaging confirmed that heated perfusate dispersed progressively and uniformly across the chamber bath within 3 min of heater activation, with no evidence of stagnant zones in the periphery (Fig. 4A). Thermistor measurements demonstrated that bath temperature reached the 36℃ target setpoint within approximately 3 min and remained stable throughout the 9-minute observation period (Fig. 4C). A transient temperature overshoot above the target was observed during the initial 1 to 2 min following heater activation, which resolved within the equilibration period. It is noted that the colorimetric scale of the infrared images in Fig. 4A denotes relative thermal distribution (HIGH/LOW) rather than absolute temperature, as infrared sensors measure surface radiance at the fluid-air interface and not bulk perfusate temperature; all absolute thermometric data reported herein were obtained exclusively from the calibrated thermistor assembly.  

**Fig. 5:**
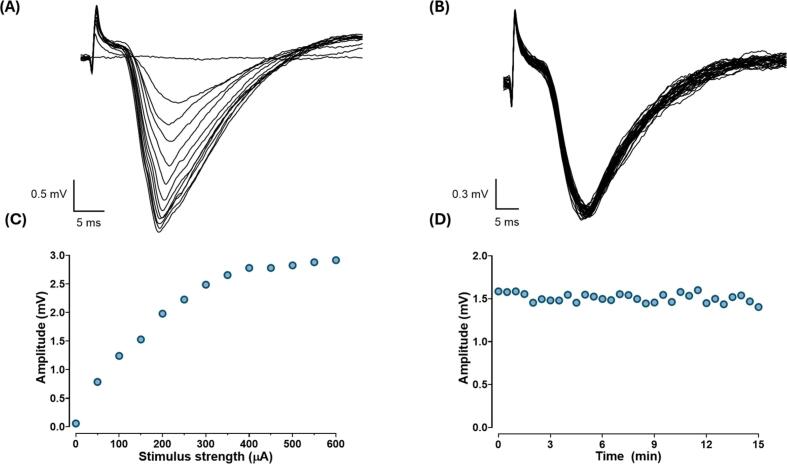
**Validation of the 3D-printed recording chamber via high-fidelity field potential recordings in hippocampal CA1.** (A) Representative fEPSP traces recorded from the stratum radiatum at incremental stimulus intensities (0 ∼ 600 μA, 30-second intervals), illustrating graded synaptic recruitment across the full stimulation range. Scale bar: 0.5 mV, 5 ms. (B) Superimposed consecutive fEPSP traces acquired during a 15-minute baseline period at a fixed stimulus intensity (60 μA, 15-second intervals), demonstrating waveform morphology consistency across sweeps. Scale bar: 0.3 mV, 5 ms. (C) Input-output curve depicting the absolute fEPSP amplitude(mV) as a function of stimulus strength(μA), showing a linear-to-saturating response profile. (D) Temporal stability of absolute fEPSP amplitude(mV) over the 15-minute baseline period, confirming robust signal maintenance with minimal inter-sweep variability.

***Electrophysiological validation (*** Fig. 5 ***):***

To assess signal quality, fEPSPs were recorded in the stratum radiatum of hippocampal area CA1 following stimulation of the Schaffer collateral-commissural pathway in CA3 in acute hippocampal slices prepared from adult male C57BL/6N mice (Fig. 3C). Input-output relationships were characterized by delivering single stimulation pulses at intensities ranging from 0 to 600 μA at 30-second intervals, with two consecutive responses averaged per intensity to yield individual data points (Fig. 5A and C). Baseline stability was assessed by delivering test pulses at 60 μA at 15-second intervals over a 15-minute period, again averaging two consecutive responses per time point (Fig. 5B and D).

The input–output relationship exhibited a consistently graded, monotonic increase in fEPSP amplitude with increasing stimulus intensity, scaling from near-zero at sub-threshold intensities to a stable plateau at supra-maximal intensities (Fig. 5A and C). Baseline fEPSP amplitudes remained stable throughout the 15-minute recording period, with minimal inter-sweep fluctuation (Fig. 5B and D).  

**Capabilities and limitations:**

• The transient temperature overshoot observed during the initial warm-up period is attributable to the feedback response kinetics of the temperature controller and is not a property of the chamber itself; it is effectively mitigated by pre-activating the perfusion heater for a minimum of 10 min prior to slice transfer. The monotonic, graded input–output relationship observed across the full stimulus range (0–600 μA) is consistent with the expected synaptic recruitment kinetics of the Schaffer collateral-CA1 pathway, confirming that signal integrity is maintained without saturation artifacts. The 15-minute baseline stability demonstrated here satisfies a critical prerequisite for long-term synaptic plasticity protocols, including LTP and LTD induction paradigms. These combined findings indicate that the platform provides a mechanically and electrically stable recording environment free from vibration artifacts, electromagnetic interference, or thermally induced signal drift.
• A direct side-by-side comparison of recording performance between this platform and a commercial recording chamber was not conducted, as an equivalent commercial system was not available in our laboratory. The primary aim of this work is not to demonstrate superiority over existing commercial products, but to establish that a freely reproducible, 3D-printed platform can achieve physiologically valid recording conditions at a fraction of the cost. The validation data presented here, encompassing stable temperature maintenance, appropriate perfusate distribution, and high-quality fEPSP recordings, are intended to demonstrate functional adequacy rather than direct equivalence to any specific commercial system.
• The platform supports stable submerged extracellular field potential recordings from acute brain slices with a signal quality comparable to that observed in commercial recording chambers, at a total material cost below 1.30 USD and a total PLA filament consumption of approximately 22.45 g.
• The recording chamber accommodates continuous perfusion at a validated flow rate of 2 mL/min and achieves stable temperature equilibration at the 36℃ target setpoint within approximately 3 min when used with a standard in-line heater and temperature controller.
• The integrated right-angle bracket within the chamber body anchors the Ag/AgCl ground electrode wire, maintaining continuous submersion within the bath solution and preventing flotation above the surface, thereby ensuring a stable electrical reference throughout the recording session.
• The modular three-component architecture permits independent replacement of the recording chamber, positioning stage, or suction assembly in the event of mechanical failure or biological contamination, without requiring full system reconstruction.
• The platform is designed as a passive fluid-handling interface with no active coupling to manufacturer-specific hardware. The perfusion inlet and outlet accept standard laboratory tubing and are compatible with any gravity-driven or pump-driven perfusion system. The open bath geometry imposes no constraints on electrode manipulator, amplifier, or digitizer selection, enabling straightforward integration into existing electrophysiology workstations regardless of manufacturer. All components can be reprinted on-demand using any consumer-grade FDM printer, enabling immediate replacement without procurement delays or additional cost.
• Assembly requires no specialized tools, adhesives for the chamber seal, or post-print processing beyond press-fitting of standard hardware nuts, reducing technical overhead and enabling rapid maintenance.
• The platform has been validated specifically for extracellular field potential recordings. Whole-cell patch-clamp recordings typically require transmitted infrared light delivered from below through a condenser for IR-DIC visualization of individual neurons; however, the opaque PLA positioning stage blocks the optical path from below, rendering this system incompatible with standard upright IR-DIC microscopy. A modified or alternative transparent stage would be required to support patch-clamp configurations
• The system was validated at a single perfusion rate of 2 mL/min. Flow uniformity under varying perfusion rates has not been systematically characterized, and formal computational fluid dynamics analysis was beyond the scope of this study.
• FDM-printed PLA components may exhibit dimensional variability between printers and filament batches. Users are advised to verify cover glass seating and nut recess dimensions after initial printing and to re-print with adjusted tolerances if assembly fit is insufficient.

## Ethics statements

All animal experiments were conducted in accordance with the ARRIVE guidelines and carried out in accordance with the guidelines of the Dankook University Ethics Committee for Care and Use of Laboratory Animals (approval number DKU-24-052) and the National Institutes of Health guide for the care and use of laboratory animals (NIH Publication No. 80-23, revised 1978). Male C57BL/6N mice were used exclusively in this study; the influence of sex on the outcomes of this study was not assessed, as the experimental objectives focused on validating the performance characteristics of the recording hardware rather than investigating sex-dependent physiological parameters.

## CRediT authorship contribution statement

**Younsoo BYUN:** Writing – review & editing, Writing – original draft, Visualization, Validation, Software, Methodology, Investigation, Formal analysis, Data curation, Conceptualization. **Hyunjun Noh:** Software, Methodology, Conceptualization. **Sung-Han Rhim:** Supervision, Resources, Project administration, Funding acquisition. **Jihyun Noh:** Writing – review & editing, Supervision, Resources, Project administration, Funding acquisition.

## Declaration of competing interest

The authors declare that they have no known competing financial interests or personal relationships that could have appeared to influence the work reported in this paper.
